# Intra- and Inter-Day Element Variability in Human Breast Milk: Pilot Study

**DOI:** 10.3390/toxics10030109

**Published:** 2022-02-25

**Authors:** Kenta Iwai, Miyuki Iwai-Shimada, Kaname Asato, Kunihiko Nakai, Yayoi Kobayashi, Shoji F. Nakayama, Nozomi Tatsuta

**Affiliations:** 1 Health and Environmental Risk Division, National Institute for Environmental Studies, Tsukuba 305-8506, Japan; iwai.kenta@nies.go.jp (K.I.); iwai.miyuki@nies.go.jp (M.I.-S.); kobayashi.yayoi@nies.go.jp (Y.K.); 2 Department of Development and Environmental Medicine, Tohoku University Graduate School of Medicine, Sendai 980-8575, Japan; asatok@med.tohoku.ac.jp (K.A.); nakai-k@tokaigakuen-u.ac.jp (K.N.)

**Keywords:** breast milk, elements, intra-day, inter-day, ICP-MS, infant exposure, toxic elements, foremilk, hindmilk

## Abstract

For infants in the first months of life, breast milk is a complete source of nutrition; however, it can also contain elements that are harmful to the infant. It is therefore critical for infant health to characterize breast milk. The aim of this study was to determine the intra- and inter-day variation of elements in breast milk, for which there is currently limited information, as a pilot study for a larger study. Firstly, we developed a simple and robust analytical method for the determination of multiple elements in breast milk. It was accurate (accuracy ranged from 98% to 107%) for measurement of 26 elements in breast milk by quadrupole inductively coupled plasma-mass spectrometry. Intra- and inter-day variation of elements, protein, and fat in breast milk was determined by analyzing breast milk collected from 11 women at 12 sampling points over three days and calculating intraclass correlation coefficients. Intraclass correlation coefficients showed that while some elements were consistent across time points (e.g., Sr, Ca, and Cu), others showed very high variability (e.g., As, Cd, and Ni). Correlation analyses between elements in breast milk showed strong relationships between those including Fe and Mo, Ca and Sr, and Cd and Fe.

## 1. Introduction

Breast milk is a complete source of infant nutrition in the first few months of life. It is a complex biological matrix consisting not only of nutrients (proteins, fats, carbohydrates, minerals, and vitamins) [1], but also other important components, including antibodies, enzymes, growth factors, hormones, cytokines, exosomes, and stem cells [2,3]. However, breast milk may also contain potentially harmful components, since some chemicals taken in by the mother (ingested, inhaled, transdermal, and injected) pass into breast milk. For example, persistent organic pollutants, polychlorinated biphenyls and poly- and perfluoroalkyl substances [4,5], pharmaceuticals [6]⁠, pesticides [7], and toxic elements [8,9] have been detected in breast milk. There are concerns about the potential impact of these chemicals and toxic elements on infants, although these effects have not been fully elucidated. Since infancy is a period of rapid development and growth, and infants have an immature digestive system and blood-brain barrier, they are as susceptible to adverse toxin responses as fetuses.

Inadequate maternal intake of some essential nutrients and elements necessary for infant development may also affect breast milk quality; the optimal daily intake of essential trace elements for infants is still under discussion. However, as well as essential elements being an important factor in development, infants are vulnerable to some toxic elements such as arsenic, lead, and mercury. In recent years, these elements have been implicated in developmental disabilities, including autism spectrum disorders [10,11]. Therefore, it is necessary to monitor the concentration of toxic elements to which infants are exposed.

Breast milk is an easily available sample for monitoring of chemical exposure in infancy, since it can be collected in a relatively non-invasive manner. By analyzing the essential and non-essential elements from abundant to trace levels in breast milk, the nutritional status of breast milk and the contamination status of known or potential toxic elements can be determined. Inductively coupled plasma-mass spectrometry (ICP-MS) is a robust analytical technique that can measure multiple elements simultaneously over a wide dynamic range. Since it can analyze even crude biological samples with high reproducibility, its performance is proven in a wide variety of specimens [12,13]. A method that can measure multiple targets simultaneously with a single specimen is expected to play an increasingly important role in investigating the causes of diseases in which there is complex interplay between many factors.

Although many papers have reported a multi-element analysis of breast milk [14], there is no centralized database due to differences in collection times, pre-treatment methods, and analysis protocols. There is also a risk of over- or underestimating the level of infant exposure depending on the sampling method, especially when breast milk elements with large intra- or inter-day variations are being studied. Therefore, the aim of this study was to develop a sampling method that can be applied to epidemiological studies. In this study, a pilot study was performed to determine the intraday variation of each element, the three-day interday variation, the difference between fore- and hindmilk, and the difference between the left and right breast. Furthermore, we measured fat content and protein concentration, which are basic nutritional components of breast milk, and performed statistical analyses to determine correlations between elements and macronutrients. Our results provide sampling methods to estimate levels of infant element exposure and provide data on the half-life, exposure profile, and chemical properties of each element in breast milk.

## 2. Materials and Methods

### 2.1. Reagents and Standard Solutions

Ultrapure water was purchased from FUJIFILM Wako Pure Chemical Corporation (Osaka, Japan) and used for the preparation of samples, standards, and solutions. All solutions were prepared in polytetrafluoroethylene bottles that were precleaned by soaking in a 5% nitric acid bath overnight or longer.

A series of standard solutions were made in a matrix-matched manner. The standard solution ranged from 0.005 to 160 μg/L for mercury (Hg); 0.02 to 640 μg/L for silver (Ag), arsenic (As), barium (Ba), beryllium (Be), cadmium (Cd), cobalt (Co), caesium (Cs), gallium (Ga), lithium (Li), manganese (Mn), molybdenum (Mo), nickel (Ni), lead (Pb), rubidium (Rb), antimony (Sb), selenium (Se), tin (Sn), uranium (U), and vanadium (V); 0.02 to 16,000 μg/L for bromine (Br), copper (Cu), rubidium (Rb), and zinc (Zn); and 0.02 to 640,000 μg/L for calcium (Ca). A calibration curve of up to 640 μg/L (or up to 160 μg/L in Hg) was prepared by adding each standard solution of Be (Sigma-Aldrich, St. Louis, MO, USA), Br (FUJIFILM Wako Pure Chemical Corporation), and Hg (FUJIFILM Wako Pure Chemical Corporation) to a XSTC-622 (SPEX CertiPrep, Metuchen, NJ, USA). Br (FUJIFILM Wako Pure Chemical Corporation), Ca (FUJIFILM Wako Pure Chemical Corporation), Cu (FUJIFILM Wako Pure Chemical Corporation), Rb (FUJIFILM Wako Pure Chemical Corporation), and Zn (FUJIFILM Wako Pure Chemical Corporation) were used to adjust the standard solutions for concentrations higher than 640 μg/L. Final concentrations of 0.12% L-cysteine (Sigma-Aldrich) and 0.56% hydrochloric acid (HCl) (FUJIFILM Wako Pure Chemical Corporation) were added to all standard solutions. These standard solutions were prepared for each measurement. A concentration of at least six points was applied to the calibration curve to cover all analytical values. The calibration curve was performed by considering the concentration of each element in breast milk.

An alkaline dilution solution was made of 4% tetramethyl ammonium hydroxide (FUJIFILM Wako Pure Chemical Corporation) and 0.1% 4H (EDTA-free acid) (Dojindo Laboratories, Kumamoto, Japan). A detergent solution consisted of 4% 2-butanol (FUJIFILM Wako Pure Chemical Corporation) and 0.1% polyoxyethylene (10) octylphenyl (FUJIFILM Wako Pure Chemical Corporation), which was fortified with indium (In), rhodium (Rh), tellurium (Te), thallium (Tl), and yttrium (Y) as internal standards (IS) (SPEX CertiPrep) to make a final concentration of 10 ppb.

### 2.2. Study Participants and Breast Milk Collection

The medical ethics committee of the Tohoku University Graduate School of Medicine and National Institute for Environmental Studies approved the study protocol. Informed consent was obtained from all individual participants included in the study. Breast milk was collected from all 11 mothers who were recruited. The age of the mothers ranged from 29 to 40 years, and the infants were three to five months old. All mothers and their infants were healthy, and infants were able to suck breast milk by themselves. Breast milk samples were collected by hand squeezing on three consecutive days: day 1, fore- and hindmilk in the morning (8:00–10:00), afternoon (11:00–13:00), and evening (18:00–20:00); day 2, fore- and hindmilk from the left and right breast in the morning (8:00–10:00); and day 3, fore- and hindmilk in the morning (8:00–10:00). Thus, with a total of 12 timepoints, we collected 132 samples of breast milk. The samples were frozen before transportation and collection, and then stored at −80 °C until analysis.

### 2.3. Sample Preparation

Breast milk samples, a standard reference material (SRM 1953; National Institute of Standards and Technology (NIST)), and sample for method detection limit (MDL) measurement were brought to room temperature by letting them sit at room temperature and gently swirling to avoid frothing, before 100 µL was aliquoted into a 15 mL MetalFree^®^ centrifuge tube (Labcon, Petaluma, CA, USA) and accurately weighed. To this sample, 3.9 mL of the alkaline dilution solution was added before samples were vortex-mixed and microwaved using the microwave digestion system MARS 6 (CEM Corporation, Matthews, NC, USA). Microwave conditions are described in Table S1. The power of the microwave was determined so as not to exceed the durability of the tube. After bringing the treated sample to room temperature, 4 mL of the detergent solution containing the internal standard was added and mixed by inverting the sample before the sample was visually examined to ensure that there was no particulate matter (Figure S1).

### 2.4. Protein Assay and Fat/Calorie Measurement

Protein content in breast milk was determined by Bradford protein assay (Bio-Rad Laboratories, Inc. Hercules, CA, USA). Breast milk samples were diluted with saline (Otsuka Pharmaceutical, Tokyo, Japan) and aliquoted into a microplate. A plate reader, Infinite M Plex (Tecan Group Ltd., Männedorf, Switzerland), was used and the assay was performed according to the manufacturer’s protocol. The calorie and fat content of breast milk was measured with the Creamatocrit Plus™ Breast Milk Fat Analyzer (EKF Diagnostics, Cardiff, UK) following the manufacturer’s protocol.

### 2.5. Instrument Analysis

A triple quadrupole inductively coupled plasma-mass spectrometer (ICP-MS/MS) (Agilent™8900 model, Agilent Technologies, Santa Clara, CA, USA) was used to analyze elements in breast milk. A continuous sample introduction system consisted of an auto sampler, SPS 4 Auto sampler (Agilent Technologies); a MicroMist nebulizer; and a quartz torch with a 2.5 mm diameter injector and a Shield Torch system. Detailed settings are shown in Table S2. The same solution used to make a blank sample was used to wash the sample probe. The IS results of each element are noted in Table S3. When measuring breast milk samples, the batch included a blank and the SRM analysis.

### 2.6. Quality Control

A linear regression was applied to construct a calibration curve, and the fitting was evaluated by a coefficient of determination (R^2^) for every batch of samples. The batch included solvent blanks, standard curve solutions with more than nine points, reference materials (NIST SRM 1953), and breast milk samples. The MDL was obtained using data from seven replicates of standard analysis that were assumed to have approximately the minimum concentration of the calibration curve [15]. The following formula was used to calculate the MDL:

MDL = 2 × s × *t*_(n − 1, 0.05)_

where *t*_(n−1, 0.05)_ represents Student’s *t* value at an α level of 0.05 with n − 1 degrees of freedom, and s represents the standard deviation (SD).

The lower limit of quantification (MQL) of the analytical method was set at 10 times the SD used to calculate the MDL:

MQL = 10 × s

### 2.7. Potential Covariates

We collected data on sociodemographic characteristics, environmental exposure, and lifestyle from self-administrated questionnaires. The mothers’ characteristics included the following: age, body mass index (BMI) before pregnancy, smoking or passive smoking, Seafood consumption and frequency in the past three months. The children’s characteristics included the following: sex (male/female), age in months, and birth weight. The daily consumption of seafood was calculated from the amount and frequency of seafood consumption (Table 1).

### 2.8. Statistical Analysis

Since element concentrations were not normally distributed, the Mann-Whitney U test was performed to examine differences in element concentrations between left and right breasts, and the difference between fore- and hindmilk was examined with a paired *t*-test using a log-transformed element, fat, and protein concentrations. The relationship between passive smoking, seafood consumption and each factor were analyzed by Spearman’s rank correlation coefficient. To examine the intraday variation, the differences between the three groups of morning, noon, and evening were carried out separately for fore- and hind milk. The Steel-Dwass test was employed to compare differences between morning and noon and between morning and evening. Intraclass correlation coefficients (ICCs) were calculated after log-transforming the concentrations of elements, fat, and protein. Approximately 10% of the samples had Co concentrations below the MDL, and thus values were substituted with MDL/√2 [16]. ICC (A,1) was performed according to Liljequist et al. [17] and computed using the irr package in R (R Core Team, 2020). ICCs were evaluated based on the guidelines proposed by Koo and Li [18]: “poor” for less than 0.5, “moderate” for between 0.50 and 0.75, “good” for between 0.76 and 0.90, and “excellent” for greater than 0.90. Since the content of fat was significantly different between fore- and hindmilk (*p* < 0.001), we calculated ICCs by dividing the data into fore- and hindmilk.

Spearman’s rank correlation coefficient (*ρ*) was computed for each analytical value of the 19 factors (17 elements, fat, and protein) for which ICC was calculated. JMP 16.1 (SAS Institute Inc, Cary, NC, USA) was carried out except for ICC calculation. The significance level was set at *p* < 0.05.

## 3. Result and Discussion

### 3.1. Method Performance Characteristics

#### 3.1.1. Pre-Treatment

ICP-MS analysis of biological samples such as blood, serum, urine, and breast milk using direct alkaline dilution is a powerful method for multi-specimen, multi-element analysis [19,20]. There are several methods of pre-treatment for breast milk [13], but we attempted the alkaline dilution method to efficiently degrade breast milk, which is a fat-rich matrix. Alkaline pre-treatment is also suitable for mercury and elements that are easily vaporizable in acidic conditions. Compared with acid pre-treatment, alkaline pre-treatment does not require high temperatures to degrade fat. Considering the characteristics of matrixes, we attempted to develop a method suitable for analyzing multiple elements in breast milk simultaneously and at high throughput. We did not apply this method to breast milk directly, since fat remains only after alkaline dilution treatment. Hence, after adding the alkaline dilution solution (tetramethyl ammonium hydroxide and EDTA) to breast milk, we treated it by microwave, followed by the addition of a detergent solution, and mixed it by inverting. No obvious suspended matter was observed after this treatment, and the addition of a small amount of cysteine and HCl to the standard solution stabilized the measurement results of Hg at low concentrations. The conditions shown in Table S1 are suitable for microwave processing of breast milk samples and do not exceed the durability of the tube. Microwave processing is an efficient pre-treatment method, since 84 samples can be performed in a single run. Due to the use of disposable tubes at relatively low temperatures under alkaline conditions, consideration of mercury vaporization and contamination was not required. This pre-treatment was simple, was completed in a single tube, did not result in adsorption of elements by the tube, and was undertaken on more than 100 samples overnight. We checked the blanks and confirmed that they were below the MDL and that the contamination of the tubes was very low.

#### 3.1.2. ICP-MS/MS Conditions

When carrying out analysis using the Agilent 8900 ICP-MS/MS, collision/reaction cells can be used to remove spectral interferences. For some of the elements that are prone to interference, the method was run with oxygen and ammonia cell gases, but the lowest MDL and stability were obtained with helium as cell gas; further verification of the method is necessary to measure elements that are susceptible to interference, such as titanium. The gas mode and integration time of all the elements measured and the information of the internal standard elements used are shown in Table S2.

#### 3.1.3. Precision, Accuracy, and Robustness

First, we calculated the linearity (R^2^) of the standard curves and MDL and MQL for all elements. The R^2^ of all the elements ranged from 0.9994 to 1.0000, and most were above 0.9999. The MDLs calculated from the measurement conditions of this study were as low as possible except for Ca and Mg (Table 2). Since Ca and Mg were abundant in the breast milk samples, the MDL was not a concern in this study.

In addition, the accuracy of the analytical method was assessed by analyzing SRM 1953, a reference material for breast milk, purchased from the US NIST (Table 3). Because there are no standard reference materials of breast milk with certified values for many elements, to ensure robustness and reliability, and to confirm contamination, the SRM 1953 was analyzed using a different instrument in a different laboratory.

Values for most of the target elements were approximate to those obtained by another company (described as Company A). However, the analytical value for Ga was very different and higher. The value of ^69^Ga was applied in this measurement, and it is highly possible that the value was high due to the interference of ^138^Ba^2+^. The Ga value is excluded in the following analysis because the monitor of ^71^Ga, another mass, was not performed. The measurement of Ga in breast milk is a future issue. The level of Mn was above the certified value, which has been previously indicated and attributed to contamination from long-term storage in SRM tubes [21]. We also analyzed blank samples and the SRM along with the breast milk samples when we measured them to evaluate the accuracy and cross-contaminations.

### 3.2. Toxic Element Levels and Fat and Protein Content in Breast Milk

We adopted the conditions used above, where we could determine a large variety of elements in a stable and repeatable manner, for the analysis of breast milk samples (*N* = 132) obtained from participants. The results are summarized in Table 4. This was the first report to obtain ICCs from 11 participants’ breast milk collected under 12 diverse conditions. Although several papers have used the ICP-MS analysis of breast milk, few methods have been reported for simultaneous multi-element measurement with Hg [22]. Monitoring the intake of Hg, which is neurotoxic, especially during the critical and sensitive period of neurodevelopment, is crucial. Although it is difficult to evaluate this analysis because it was based on samples obtained from 11 people, the analytical values for many elements were close to those reported in previous studies. The concentrations of Hg and Cd were at moderate exposure levels compared with values from 14 countries or regions previously summarized by Vollset et al. [23]. By contrast, As levels in breast milk were higher, with median values comparable to values in Bangladesh, a highly contaminated area, and maximum values were much higher [24]. Since Japanese people ingest most arsenic from seafood and seaweed, it is likely that measured levels came from organic arsenic, such as arsenobetaine, which has relatively low toxicity; however, confirmation of this may be necessary in future due to concerns about potential toxicity [25]. Although there are few speciation analyses of arsenic in breast milk, speciation analyses of arsenic in breast milk have been reported in areas with inorganic arsenic contamination of drinking water. Most of the arsenic in breast milk is inorganic arsenic, suggesting that arsenic metabolized by methylation in the maternal body may be difficult to release from breast milk [26]. In the future, speciation analysis of arsenic in breast milk is a major issue.

The content of fat and protein in the breast milk of 132 samples is also summarized in Table 5. Compared with previous reports, the protein content was similar, but fat was higher: this result may be because we included foremilk samples, which tend to have higher fat content [27].

### 3.3. Relationship between Elements in Breast Milk, Passive Smoking, and Seafood Consumption

There were no smokers among the 11 participants in this study, and there were four people who were passive smoking (Table 1). No statistical difference was identified between Cd or Pb and passive smoking. All participants who answered passive smoking reported that smokers in their households smoked in isolated areas. Therefore, the impact was considered to be limited.

The relationship between the calculated amount of seafood consumed per day and the concentration of the elements in breast milk was examined; correlations were found with Hg (0.531), Br (0.463), Se (0.409), and As (0.375). These four elements are elements typical of those contained in seafood [28].

### 3.4. Difference between Foremilk and Hind Milk

In determining the intra- and inter-day variation of factors in breast milk, we showed the difference of elements (Table 6), fat and protein contents in between fore- and hindmilk (Table 7). The intra-day variation is also summarized in Tables S4 and S5. As previously reported, differences in fat and protein content can be seen [29]. There were also differences in the amount of Mn, Fe, Cu, As, Se, Mo, and Cd (*p* < 0.001). To account for these differences, ICC was calculated separately for fore- and hindmilk. It is also important to standardize the sampling timing when designing a sampling protocol. Some elements vary between fore-and hindmilk and should be taken into consideration when estimating infant exposure. As for the intraday variation, each morning value was compared as the standard; however, there was no significant difference, probably because of the small population. (Tables S4 and S5).

### 3.5. ICC Calculation

Intra- and inter-day variation was evaluated by obtaining ICCs separately for four conditions (Table 8). ICCs were calculated for fore- and hindmilk, but no elements had significantly different values between fore- and hindmilk, except for Co. The essential elements Ca, Cu, and Zn were “good” in all conditions (>0.76); Ca and Cu are known to have low inter-day variation, which supports the results [30]. The highest value element was Sr, a non-essential element, with an “excellent” ICC (>0.95) in all conditions. This result is likely related to the fact that Sr has similar kinetics in the body to Ca [31], which is abundant in breast milk. The ICCs of Rb and Br also tended to be high. Although there is no clear evidence that it is an essential element, organisms readily absorb Rb, while recent reports suggest that Br may function as an essential element [32,33]. As and Cd, both toxic elements, had high inter-day variation, resulting in low ICCs (As, 0.18 and 0.33; Cd, 0.36 and 0.25 for fore- and hindmilk, respectively). Because As is rapidly eliminated after ingestion, arsenic concentrations in breast milk would tend to be more variable and affected by seafood consumption. Japanese people consume Cd on a daily basis, mainly from seafood and rice, with the concentration of Cd in seafood higher than that in rice [34]. The intake of these foods may, therefore, have affected the variation in As and Cd concentration in breast milk. ICC analyses indicated that the sample size for good reproducibility of As or Cd concentrations in breast milk required at least 3 or 4 days’ sampling for risk assessment of infants through breastfeeding. Fat and Fe also showed high intra-day variation, as in previous reports (ICCs for fat, 0.33 and 0.38; Fe, 0.1 and 0.38 for fore- and hindmilk, respectively) [30]. The ICCs for Se, Mo, and Co, which are essential trace elements, were rated “good” or “moderate” for Se and Mo, but “poor” for Co in three of the four conditions. Ni had particularly high intra- and inter-day variation (ICCs for intra-day, 0.00 and 0.25; inter-day, 0.03 and 0.04 for fore- and hindmilk, respectively), suggesting it may be difficult to assess infant exposure to Ni from breast milk exclusively. The data on Hg are not shown here and will be published separately. Additionally, we investigated differences in breast milk between the left and right breasts: a slight difference was observed only in Mo (*p* = 0.046). It is unclear why the difference was observed, but it may be due to the small number of participants. The ICCs obtained in this study are based on the limited information obtained from the 11 participants in the pilot study; however, we measured the most elements under the conditions adequate to calculate the ICC.

### 3.6. Correlation between Each Element and Amount of Fat and Protein

**Figure 1:**
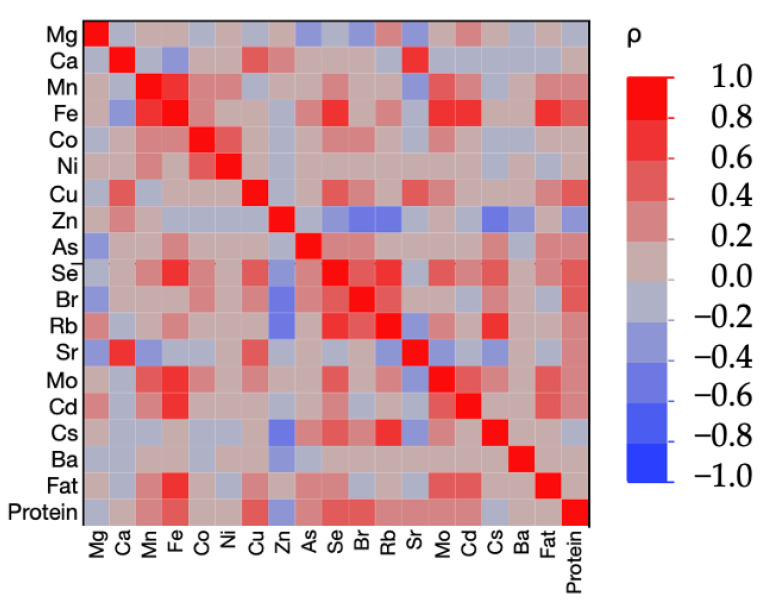
Heatmap of Spearman correlation coefficients (*ρ*) among 17 elements, fat, and protein. Positive correlations are shown in red and negative correlations are shown in blue.

We calculated Spearman’s rank correlation coefficient (*ρ*) to determine the correlation between 19 factors for which the ICC was calculated (Figure 1). The strong correlation between Fe and Mo (0.75) suggests an interaction between these elements, possibly via xanthine oxidase, which releases Fe from ferritin [35]. Patients with Fe deficiency anemia are reported to have low Mo levels in the blood, and simultaneous administration of Mo and iron improves the effect of Fe supplementation on anemia during pregnancy [36]. We also observed a correlation between Ca and Sr (0.67), both of which had high ICCs (Table 8) and have similar biokinetics [31]. Cd, a toxic element, was correlated with Fe (0.66). The relationship between Cd and Fe has been discussed in several papers: Fe deficiency is associated with increased Cd uptake [37,38], suggesting that Fe may facilitate the release of Cd into breast milk. By contrast, since Fe deficiency in infants is not associated with Cd levels in the blood [39], Fe may be secreted with Cd, but not readily absorbed if iron is in sufficient supply. Levels of Cd in breast milk were lower in mothers with older infants, confirming the theorized passage of Cd from the mother to the child, especially during the early lactation period [40]. Thus, health risks for younger infants should be considered. Data on mercury are not shown here and will be published separately.

### 3.7. Limitations

There were some limitations to this study. Firstly, the sample size was small with 11 participants, so we were not able to target elements that were mostly below the MDL. In addition, the source of exposure to elements was unknown due to the limited information on the participants’ diet and other environmental factors.

## 4. Conclusions

In order to investigate infant exposure to elements, an optimized sampling protocol for breast milk is required. In this study, we first developed a simple and robust method for multi-element analysis of breast milk in an attempt to determine the ICC of elements in breast milk. The method uses only 100 μL of liquid breast milk, with pre-treatment of 84 samples taking only a few hours and the measurement of more than 100 samples possible in an overnight analysis. Breast milk samples collected from 11 participants as a pilot study under 12 diverse conditions not previously reported were analyzed, and ICCs of protein and fat contents and element were obtained. To estimate exposure to the toxic elements As and Cd through breast milk, at least three to four days of sampling are needed. A larger survey to determine the ICCs of Pb and other elements of concern for health effects, which could not be calculated in this study, are required. The correlations between elements and with macronutrients from this study may provide fundamental data to help understand the mechanisms of exposure to elements through breast milk.

## Figures and Tables

**Table 1:** Characteristics of the participants population.

	Mean ± SD	Median	IQR	%
Mother				
Maternal age	33.1 ± 3.6			
Pre-pregnancy BMI	21.7 ± 2.9			
Passive smoking (%, Yes)				36.4%
Seafood consumption (g/day)		7.5	5.6	
Child				
Child sex (%, male)				63.6%
Child’s age in months				
3 months				36.4%
4 months				45.5%
5 months				18.2%
Birth weight (g)	3045 ± 313.9			

SD: standard deviation, IQR: interquartile range, BMI: body mass index.

**Table 2:** Summary of method performance parameters.

	Linearity (R^2^)	MDL (ng/mL)	MQL (ng/mL)
Li	0.9999	0.054	0.140
Be	0.9999	0.0082	0.021
Mg	0.9998	31	79
Ca	0.9998	318	818
V	0.9998	0.068	0.174
Mn	0.9999	0.24	0.62
Fe	1.0000	65	168
Co	0.9998	0.039	0.10
Ni	0.9998	0.96	2.5
Cu	1.0000	1.3	3.4
Zn	1.0000	1.5	3.8
Ga	0.9994	0.11	0.19
As	0.9999	0.071	0.18
Se	0.9996	0.43	1.1
Br	1.0000	1.7	4.4
Rb	0.9999	0.28	0.71
Sr	0.9999	0.061	0.16
Mo	0.9999	0.12	0.31
Ag	0.9999	0.023	0.059
Cd	0.9997	0.094	0.24
Sb	1.0000	0.049	0.13
Cs	0.9998	0.11	0.29
Ba	0.9999	0.052	0.13
W	0.9999	0.054	0.14
Hg	1.0000	0.040	0.10
Pb	1.0000	0.078	0.20
U	1.0000	0.019	0.049

MDL: method detection limit, MQL: method quantification limit.

**Table 3:** Analysis results for National Institute of Standards and Technology standard reference material (1953) in our laboratory, another institution, and published data.

		Our Study		Company A		Published Paper
	Certified Value (ng/g) (Acceptable Range)	Mean ^a^ (ng/g)	RSD (%)	Mean ^b^ (ng/g)	RSD (%)	Median (ng/g)
Li		3.0	1.7	2.55	1.2	
Be		0.014	17	<0.041		
Mg	32,400 (32,200−32,600)	34,900	3.4	32,400	2.3	35,000
Ca	257,000 (255,000−259,000)	258,000	1.9	248,000	1.5	257,000
V		0.23	5.1	0.201	17	
Mn	40 (38−42)	64	2.0	51.3	2.7	60.5
Fe	194 (187−201)	226	1.7	198	3.3	205
Co		0.085	14	0.089	0.071	
Ni		2.39	10	1.54	8.9	
Cu	268 (265−271)	269	1.7	264	2.6	284
Zn		2226	1.4	1950	3.3	2637
Ga		1.6	0.3	0.0138	152	
As		0.23	5.0	<0.32		
Se		16	6.6	14.6	2.9	17.3
Br		1642	1.7	1300	3.4	
Rb		464	1.0	470	472	
Sr		49	0.4	48	46.9	
Mo		2.1	0.3	2.1	2.21	<PLOQ
Ag		0.054	10	0.084	0.105	
Cd		0.063	23	0.054	0.0285	
Sb		<0.049		<0.065		
Cs		2.2	8.7	1.7	1.71	
Ba		13	2.0	11	10.7	
W		<0.054		0.2	0.248	
Hg	0.101 (0.068−0.134)	0.112	7.6	0.094	0.101	
Pb		0.44	12	0.29	0.318	
U		< 0.019				

^a^*N* = 4; ^b^ *N* = 5–6. Results from the company that commissioned the analysis are shown as ‘Company A’. The results labelled ‘Published paper’ are from Dubascoux et al., 2018, and are displayed as medians. PLOQ: Practical Limits of Quantification [21], RSD: relative standard deviation.

**Table 4:** Concentrations of 27 elements in human breast milk (*N* = 132).

	MDL (ng/mL)	Detection (*N*)	Min (ng/g)	P5 (ng/g)	Median (ng/g)	P95 (ng/g)	Max (ng/g)
Li	0.054	97	<0.054	<0.054	0.10	0.26	0.47
Be	0.0082	22	<0.0082	<0.0082	<0.0082	0.013	0.027
Mg	31	132	27,007	28,438	34,015	43,151	45,204
Ca	318	132	199,161	210,063	306,797	360,005	397,557
V	0.068	51	<0.068	<0.068	<0.068	0.14	0.45
Mn	0.24	132	1.5	1.6	3.1	7.4	10
Fe	65	132	70	96	234	543	657
Co	0.039	116	<0.039	<0.039	0.062	0.15	0.73
Ni	0.96	132	1.6	2.5	3.8	12	38
Cu	1.3	132	73	90	246	392	493
Zn	1.5	132	376	538	1011	2235	2496
As	0.071	132	0.48	0.62	1.3	7.2	24
Se	0.43	132	12	12	16	22	27
Br	1.7	132	665	742	1039	1493	1723
Rb	0.28	132	345	361	439	553	609
Sr	0.061	132	22	27	42	97	117
Mo	0.12	132	0.18	0.39	1.5	6.3	12
Ag	0.023	90	<0.023	<0.023	0.057	0.44	0.57
Cd	0.094	132	0.11	0.13	0.20	0.35	0.42
Sb	0.049	86	<0.049	<0.049	0.063	0.17	0.43
Cs	0.11	132	0.8	0.9	1.4	3.1	8.1
Ba	0.052	132	0.4	0.6	1.7	6.5	27
W	0.054	1	<0.054	<0.054	<0.054	<0.054	4.6
Hg	0.040	132	0.06	0.1	0.57	1.4	2.1
Pb	0.078	89	<0.078	<0.078	0.11	0.57	45
U	0.019	0	<0.019	<0.019	<0.019	<0.019	<0.019

MDL: method detection limit, P5: 5th percentile, P95: 95th percentile.

**Table 5:** Fat, calorie, and protein concentrations in human breast milk (*N* = 132).

	Min	P5	Median	P95	Max
Fat (g/L)	16	22	53	108	123
Calorie (kcal/mL)	519	558	850	1362	1510
Protein (mg/mL)	5.6	6.1	7.9	11	12

P5: 5th percentile, P95: 95th percentile.

**Table 6:** Difference in elements between fore- and hindmilk.

	Foremilk (*N* = 66)		Hindmilk (*N* = 66)		
	Median	IQR	Median	IQR	Paired *t*-Test
	(ng/g)	(ng/g)	(ng/g)	(ng/g)	(*p*-Value)
Li	0.1	0.14	0.097	0.091	
Be	<0.0082		<0.0082		NA
Mg	33,917	6251	34,206	6345	
Ca	304,659	64,672	309,864	69,771	
V	<0.068		<0.068		NA
Mn	2.8	1.4	3.5	2.2	<0.001
Fe	162	91	314	194	<0.001
Co	0.059	0.031	0.067	0.043	
Ni	4	3.5	3.8	2.4	
Cu	235	110	266	110	<0.001
Zn	1027	520	1005	555	
As	1.1	0.73	1.6	1.6	<0.001
Se	15	3.8	16	4.9	<0.001
Br	1021	351	1064	356	
Rb	443	89	438	84	
Sr	41	27	43	29	
Mo	1	1.1	2.1	1.5	<0.001
Ag	0.049	0.1	0.06	0.1	
Cd	0.17	0.065	0.23	0.079	<0.001
Sb	0.062	0.05	0.064	0.037	
Cs	1.5	0.55	1.4	0.55	
Ba	1.6	2.1	1.8	2	
W	<0.054		<0.054		NA
Hg	0.47	0.46	0.63	0.59	<0.001
Pb	0.11	0.11	0.1	0.062	
U	<0.019		<0.019		NA

IQR: interquartile range, NA: not analysiss. Significant differences (<0.001) are indicated by paired *t*-test.

**Table 7:** Difference in protein and fat contents between fore- and hindmilk.

	Foremilk (*N* = 66)		Hindmilk (*N* = 66)		
	Median	IQR	Median	IQR	Paired *t*-Test
					(*p*-Value)
Fat (g/L)	43	21	66	33	<0.001
Protein (mg/mL)	7.6	2.1	8.4	2.1	<0.001

IQR: interquartile range. Significant differences (<0.001) are indicated by paired *t*-test.

**Table 8:** ICCs of elements, fat, and protein in breast milk.

ICC (A,1)				
	Intra-Day		Inter-Day	
	Foremilk	Hindmilk	Foremilk	Hindmilk
Mg	0.69	0.46	0.81	0.70
Ca	0.82	0.90	0.92	0.83
Mn	0.44	0.57	0.42	0.80
Fe	0.38	0.59	0.10	0.38
Co	0.030	0.51	0.17	0.18
Ni	0.00	0.25	0.033	0.04
Cu	0.88	0.87	0.89	0.90
Zn	0.83	0.76	0.91	0.86
As	0.69	0.83	0.18	0.33
Se	0.61	0.64	0.55	0.67
Br	0.82	0.68	0.88	0.86
Rb	0.83	0.86	0.89	0.75
Sr	0.95	0.95	0.97	0.95
Mo	0.73	0.87	0.63	0.81
Cd	0.42	0.58	0.36	0.25
Cs	0.56	0.78	0.63	0.51
Ba	0.77	0.70	0.74	0.52
Fat	0.17	0.029	0.33	0.38
Protein	0.76	0.76	0.69	0.73

ICC: Intraclass correlation coefficient, Intra-day: ICC of samples taken at three time points in one day, Inter-day: ICC samples taken at the same time on three different days.

## Data Availability

Data are unsuitable for public deposition due to ethical restrictions and the legal framework in Japan. The Act on the Protection of Personal Information (Act No. 57 passed on 30 May 2003, amended on 9 September 2015) prohibits the public deposition of data containing personal information. Ethical Guidelines for Medical and Health Research Involving Human Subjects enforced by the Japan Ministry of Education, Culture, Sports, Science and Technology and the Ministry of Health, Labour and Welfare also restrict the open sharing of epidemiologic data. All inquiries about access to data should be sent to corresponding authors.

## Supplementary Materials

The following supporting information can be downloaded at: https://www.mdpi.com/article/10.3390/toxics10030109/s1, Figure S1: Pre-treatment procedures for ICP-MS analysis of breast milk samples, Table S1: Microwave conditions, Table S2. ICP-MS settings, Table S3. Setting parameters for the ICP-MS method, Table S4. Intra-day variation of elements between fore- and hindmilk, Table S5. Intra-day variation of fat and protein contents between fore- and hindmilk.
