# Supplementary material for: Intra- and Inter-Day Element Variability in Human Breast Milk: Pilot Study
Source: Toxics. 2022 Feb 25;10(3):109. doi: 10.3390/toxics10030109 (PMC8951413; doi:10.3390/toxics10030109)
Supplement: Supplementary file 1 [file toxics-10-00109-s001.zip › toxics-1595563-supplementary.pdf]

toxics

Supplementary Materials: Intra- and Inter-Day Element Variability in Human Breast Milk: Pilot Study

Kenta Iwai, Miyuki Iwai-Shimada, Kaname Asato, Kunihiko Nakai, Yayoi Kobayashi, Shoji F. Nakayama and Nozomi Tatsuta

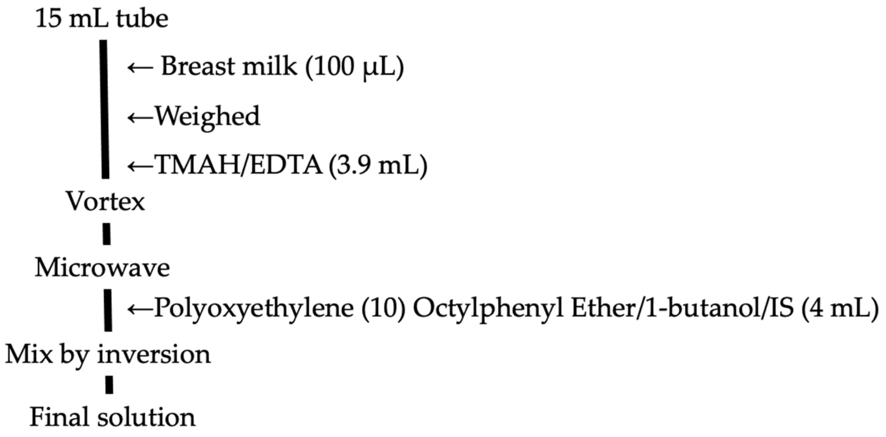

Figure S1. Pre-treatment procedures for ICP-MS analysis of breast milk samples. ICP-MS: inductively coupled plasma-mass spectroscopy, TMAH: tetramethyl ammonium hydroxide, IS: internal standerd.

Table S1. Microwave conditions.

**Power (W)**	**600**		
Ramp time (min)	5		
Step	1	2	3
Hold (min)	10	20	30
Temperature (°C)	60	70	80

Table S2. ICP-MS settings.

**Parameters**	**Setting**
RF power	1550 W
RF matching	1.20 V
Sample/skimmer cone	Nickel cones
Sampling position	8.0 mm
Nebulizer gas	1.08 L/min
Nebulizer uptake	Micromist concentric nebulizer
Option gas	-
Nebulizer pump	0.10 rps
Spray chamber temperature	2°C
Make up gas	-
Aux. gas	0.90 L/min
Plasma gas	15.0 L/min
Collision gas flow rates (He gas)	L-He: 3.5 mL/min
	H-He: 4.7 mL/min

ICP-MS: inductively coupled plasma-mass spectroscopy, RF: radio frequency.

Table S3. Setting parameters for the ICP-MS method.

	Target mass	Gas mode	Integration time/mass	Internal standard elements
Li	7	No gas	0.5	Y
Be	9	No gas	1	Y
Mg	24	L-He	0.03	Y
Ca	43	L-He	0.06	Y
V	51	H-He	1	Y
Mn	55	H-He	0.1	Y
Fe	56	L-He	0.1	Y
Co	59	H-He	0.1	Y
Ni	60	L-He	0.1	Y
Cu	63	No gas	0.1	Y
Zn	66	No gas	0.5	Y
Ga	69	L-He	0.05	Y
As	75	H-He	0.5	Te
Se	78	L-He	0.1	Te
Br	79	No-gas	0.1	Y
Rb	85	No-gas	0.05	Y
Sr	88	No-gas	0.1	Y
Mo	95	No-gas	0.1	Y
Ag	107	H-He	0.5	Rh
Cd	111	L-He	0.1	In
Sb	121	L-He	0.1	In
Cs	133	H-He	0.1	In
Ba	138	L-He	1	In
W	182	No-gas	0.1	Tl
Hg	200	No-gas	1	Tl
Pb	208	H-He	0.1	Tl
U	238	H-He	0.1	Tl
Y	89	No gas	0.01	
Tl	205	No gas	0.6	
Y	89	L-He	0.01	
In	115	L-He	0.01	
Te	125	L-He	0.1	
Y	89	H-He	0.01	
Rh	103	H-He	0.1	
In	115	H-He	0.01	
Te	125	H-He	0.01	
Tl	205	H-He	0.6	

ICP-MS: inductively coupled plasma-mass spectroscopy.

Table S4. Intra-day variation of elements between fore- and hindmilk.

	Foremilk (ng/g)			Hindmilk (ng/g)		
	Morning (*N*=11)	Afternoon (*N*=11)	Evening (*N*=11)	Morning (*N*=11)	Afternoon (*N*=11)	Evening (*N*=11)
Li	0.1	0.062	0.11	0.12	0.086	0.11
Be	< 0.0082	< 0.0082	< 0.0082	< 0.0082	< 0.0082	< 0.0082
Mg	34196	33938	31351	34679	33739	33712
Ca	297801	317211	325290	296616	308159	310641
V	< 0.068	< 0.068	0.07	< 0.068	0.087	< 0.068

Mn	2.8	2.3	2.8	3.4	4.1	3.4
Fe	163	151	166	337	376	338
Co	0.057	0.059	0.072	0.065	0.083	0.068
Ni	3.4	4.1	3.5	3.9	3.8	3.8
Cu	233	243	209	266	260	234
Zn	1057	854	956	1031	1011	861
As	1.04	1.2	1.18	1.89	1.71	1.6
Se	14	16	15	16	19	17
Br	982	1082	1023	996	1071	1051
Rb	441	448	453	449	448	470
Sr	39	41	45	39	44	44
Mo	1	1	1.4	2.1	2.7	2.3
Ag	0.059	0.06	0.048	0.076	0.108	0.062
Cd	0.16	0.17	0.21	0.24	0.23	0.24
Sb	< 0.049	0.075	0.095	0.063	0.065	0.07
Cs	1.4	1.5	1.4	1.4	1.4	1.5
Ba	1.6	1.7	1.7	1.7	2.3	1.9
W	< 0.054	< 0.054	< 0.054	< 0.054	< 0.054	< 0.054
Hg	0.64	0.42	0.6	0.75	0.77	0.71
Pb	0.1	0.13	0.12	0.13	0.13	0.12
U	< 0.019	< 0.019	< 0.019	< 0.019	< 0.019	< 0.019

Table S5. Intra-day variation of fat and protein contents between fore- and hindmilk.

	**Foremilk**			**Hindmilk**		
	**Morning (*N*=11)**	**Afternoon (*N*=11)**	**Evening (*N*=11)**	**Morning (*N*=11)**	**Afternoon (*N*=11)**	**Evening (*N*=11)**
Fat (g/L)	47	35	38	63	71	71
Protein (mg/mL)	7.5	7.6	7.1	8.4	8.2	8.6
